# The Identification and Verification of Key Long Noncoding RNAs in Ischemic Stroke

**DOI:** 10.1155/2020/2094320

**Published:** 2020-12-29

**Authors:** Houxi Xu, Jinzhi Zhang, Yuzhu Ma, Jialin Gu, Xinyue Jing, Shengfeng Lu, Xia Chen, Wenguo Yang, Yaoyao Bian, Shuping Fu

**Affiliations:** ^1^Key Laboratory of Acupuncture and Medicine Research of the Ministry of Education, Nanjing University of Chinese Medicine, Nanjing 210028, China; ^2^Affiliated Hospital of Integrated Traditional Chinese and Western Medicine, Nanjing University of Chinese Medicine, Nanjing 210028, China; ^3^School of Artificial Intelligence and Information Technology, Nanjing University of Chinese Medicine, Nanjing 210028, China; ^4^Nursing of School, Nanjing University of Chinese Medicine, Nanjing 210028, China

## Abstract

Stroke is a neurological disease with high rates of mortality and disability. The pathogenesis of stroke is acute focal injury of the central nervous system, leading to impaired neural function. Ischemic stroke accounts for the majority of cases. At present, the exact molecular mechanism of ischemic stroke remains unclear. Studies have shown that long noncoding RNAs (lncRNAs) have an important regulatory role in biological processes, participating in the regulation of transcription and affecting the processing and splicing of mRNAs. Abnormal lncRNA expression is associated with various diseases, including diseases of the nervous system. To identify and verify the key lncRNAs in ischemic stroke, we downloaded gene expression data from the National Center for Biotechnology Information Gene Expression Omnibus (NCBI GEO) and obtain differentially expressed lncRNAs, miRNAs, and mRNAs by bioinformatics analysis. Cytoscape was used to reconstruct a lncRNA-miRNA-mRNA network on the basis of the competitive endogenous RNA theory. We performed Gene Ontology (GO) and Kyoto Encyclopedia of Genes and Genomes (KEGG) pathway analyses of the mRNAs regulated by lncRNAs in the lncRNA-miRNA-mRNA network. The resulting lncRNA-miRNA-mRNA network was composed of 91 lncRNA nodes, 70 mRNA nodes, 21 miRNA nodes, and 288 edges. GO analysis and KEGG pathway analysis have shown that 191 GO terms and 23 KEGG pathways were enriched. Finally, we found that four key lncRNAs were highly correlated with ischemic stroke and could be used as potential new targets for treatment.

## 1. Introduction

Stroke is considered the second leading cause of death after ischemic heart disease and the third leading cause of disability worldwide [1]. Each year, 780,000 people experience stroke, with ischemic stroke accounting for approximately 87% of all cases [2]. However, the precise molecular mechanism of ischemic stroke is not yet known. There is an urgent need to clarify the etiological mechanism of ischemic stroke and to identify new biomarkers and therapeutic targets.

Long noncoding RNAs (lncRNAs), which are RNAs more than 200 nucleotides in length with no protein-coding capacity, have an important role in biological processes, participate posttranscriptional regulation, cell-cell signaling, and protein allosteric regulation [3, 4]. A number of related studies have shown that the processes involved in the occurrence and development of various diseases are associated with the abnormal expression of lncRNAs. For example, high expression of lncSNHG15 can promote the proliferation of cancer cells in intestinal, breast, prostate, and lung cancer [5–8]. Although there have been many studies related to lncRNAs, few studies have examined the key lncRNAs associated with ischemic stroke.

The competitive endogenous RNA (ceRNA) theory suggests that protein-coding genes, pseudogenes, and lncRNAs compete to bind to the same microRNA (miRNA) through miRNA response elements (MREs) to regulate gene expression [9]. For example, lncRNA H19, an important carcinogen in colorectal cancer, can competitively bind to miR-138 and miR-200a and regulate the expression of key genes vimentin, ZEB1, and ZEB2, thereby promoting epithelial-mesenchymal transition and thus promoting cancer progression [10]. The use of ceRNA networks to search for key lncRNAs is a new research perspective. It has been applied in many studies of diseases including cancer, abdominal aortic aneurysm, and systemic lupus erythematosus. In this study, we used a ceRNA network to search for key lncRNAs involved in ischemic stroke [11–13].

First, we downloaded the ischemic stroke gene expression datasets from the National Center for Biotechnology Information Gene Expression Omnibus (NCBI GEO) database and used bioinformatics methods to identify the differentially expressed lncRNAs (DELs), miRNAs (DEMis), and mRNAs (DEMs). Using the ceRNA theory, we constructed a lncRNA-miRNA-mRNA network to determine the functional lncRNAs in ischemic stroke, screen key lncRNAs highly related to the disease, predict their molecular regulatory mechanisms, and identify new targets for diagnosis and treatment. Finally, key lncRNAs were molecularly verified using quantitative real-time PCR (qRT-PCR).

## 2. Materials and Methods

### 2.1. Raw Data Downloaded

NCBI GEO is a public functional genomics data repository composed of high-throughput microarray and next-generation sequencing functional genome data, providing tools to help users query and download gene expression profiles [14]. Expression data for miRNAs (GSE110993), lncRNAs, and mRNAs (GSE122709) were downloaded from GEO by using wget command. The platform for GSE110993 was GPL15456 (Illumina HiScanSQ, *Homo sapiens*), which included 20 blood samples from stroke patients and 20 from healthy control subjects, respectively. The platform for GSE122709 was GPL20795 (HiSeq X Ten), which included five blood samples from stroke patients and five from healthy control subjects, respectively. In addition, hsa-miR-143-3p, which has been identified as an early diagnostic marker for acute ischemic stroke, was added to the DEMis [15].

### 2.2. Identification of DEMis, DELs, and DEMs

Differential expression analysis of lncRNA expression data was performed using the DESeq2 and edgeR packages in the R language [16, 17]. The criteria for differential expression of lncRNAs were FDR < 0.05 and log2 | fold change | >1. Other parameters of edgeR and DESeq2 use the default parameters. The intersection function in R was used to identify the common differentially expressed lncRNAs between the results of DESeq2 and edgeR analyses. The common differentially expressed lncRNAs were considered to be differentially expressed lncRNAs (DELs) in ischemic stroke. A Venn diagram was generated by the VennDiagram R package. The same method was used to obtain DEMs and DEMis of ischemic stroke.

### 2.3. Prediction of Target lncRNAs and mRNAs of DEMis

The target genes of DEMis were predicted using MiRwalk 3.0 (http://mirwalk.umm.uni-heidelberg.de), which is a comprehensive database for predicting miRNA target genes and verifying miRNA binding sites [18]. The prediction process of MiRwalk 3.0 is as follows: open the website of MiRwalk 3.0, select the miRNA prediction module, and then set the species to human and the miRNA datatype to miRbaseIDs. Finally, enter the miRNA name in the box and submit it. The screening criterion for prediction results is that TargetScan database and MiRDB database can jointly predict the target gene [19, 20]. We also used LncBase Predicted v.2 (http://carolina.imis.athena-innovation.gr/diana_tools/web/) to predict target lncRNAs of DEMis, which is a database that specifically records the interactions between miRNAs and lncRNAs [21]. The prediction process of LncBase Predicted v.2 is as follows: open the LncBase Predicted v.2 website, set the threshold to 0.7, and use the default parameters provided by the website for other parameters, and then enter the miRNA name in the miRNA module and submit it.

### 2.4. Construction of the lncRNA-miRNA-mRNA Network

The intersection function in R was used to identify the common lncRNAs between the DELs and the predicted lncRNAs of DEMis. The common lncRNAs were used to construct ceRNA networks for ischemic stroke. In the same way, we obtained the common mRNAs which were also used to construct ceRNA networks for ischemic stroke. DEMis were used to construct a ceRNA network for ischemic stroke.

Cytoscape is an open source network visualization software platform based on Java technology that is mainly used for the analysis, research, and design of complex biological networks. It can generate gene expression regulatory networks, protein interaction networks, and other aspects of the structure and hierarchy of networks. In this study, we used Cytoscape to construct and visualize a lncRNA-miRNA-mRNA network. In Cytoscape's network, our settings are as follows: the diamond nodes represent the lncRNAs, the circular nodes represent the mRNAs, and the square nodes represent the miRNAs. Red represents upregulated expression, whereas green represents downregulated expression. Other parameters of Cytoscape use the default parameters. The network is modularized according to the function of miRNA. The size of the node represents the degree of the node.

### 2.5. GO Analysis and KEGG Pathway Analysis of the lncRNAs in the ceRNA Network

ClusterProfiler is a new tool based on Gene Ontology (GO) that performs statistical analysis and visualization of functional clustering of gene clusters [22]. To predict the biological function of lncRNAs in the ceRNA network of ischemic stroke, we performed GO analysis and Kyoto Encyclopedia of Genes and Genomes (KEGG) pathway analysis on the mRNAs in the ceRNA network using ClusterProfiler and follow the methods provided by the ClusterProfiler package for GO analysis and KEGG pathway analysis. The screening conditions of GO analysis and KEGG pathway analysis were *p* value <0.05.

### 2.6. Construction of the lncRNA-miRNA-mRNA Subnetwork

Node and node degree are two key elements in a ceRNA network. We extracted all the nodes and calculated their degrees in the lncRNA-miRNA-mRNA network for ischemic stroke. We also calculated the number of first relationship lncRNA-miRNA pairs and secondary relationship miRNA-mRNA pairs. The key lncRNAs were determined based on these three factors. Cytoscape is a software platform designed for the visualization of molecular interaction networks and biological pathways, which is commonly used to construct biological networks [23]. In this study, Cytoscape was used to construct the ceRNA network of key lncRNAs. To predict the function of key lncRNAs, we conducted GO analysis and KEGG pathway analysis of the mRNAs regulated by key lncRNAs.

### 2.7. Verification of Key lncRNAs

To validate the key identified lncRNAs, we selected six blood samples from ischemic stroke patients and six blood samples from healthy control subjects for qRT-PCR molecular validation. We extracted total RNA from the blood using TRIzol reagent. Total RNA was reverse transcribed into cDNA using a PrimeScript RT reagent kit with gDNA Eraser (Takara, Dalian, China). The primers were designed using Primer BLAST. The expression levels of lncRNAs were measured using a real-time PCR system (Applied Biosystems, Foster City, CA, USA). GAPDH was used as the internal reference gene. Finally, we analyzed the data by the comparative quantitative cycle (Cq) (2^-*ΔΔ*Cq^) method. The research was approved by the Ethics Committee of the Jiangsu Provincial Hospital of Traditional Chinese Medicine. All patients provided written informed consent for research on their specimens. For detailed information regarding qRT-PCR primers of lncRNAs, please refer to Supplementary Material Table S1.

## 3. Results

### 3.1. DELs, DEMs, and DEMi Screening Results

**Figure 1:**
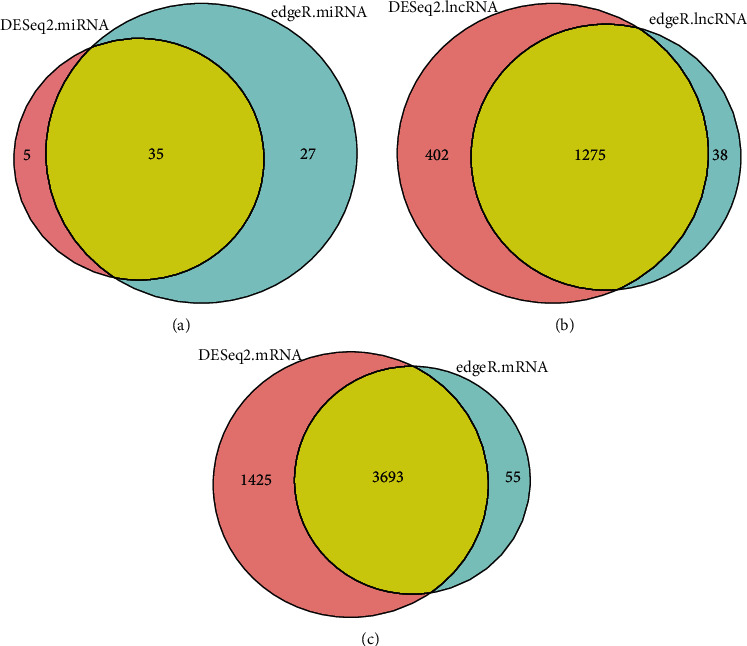
Screening results for differentially expressed lncRNAs, miRNAs, and mRNAs using DESeq2 and edgeR. The two circles in part A represent the differentially expressed miRNAs obtained using DESeq2 and edgeR, and yellow indicates the common differentially expressed miRNA; the two circles in part B represent the differentially expressed lncRNAs obtained using DESeq2 and edgeR, and the yellow part represents the common differentially expressed lncRNAs; the two circles in part C represent the differentially expressed mRNAs obtained using DESeq2 and edgeR, and the yellow part represents the common differentially expressed mRNAs.

We used the DESeq2 package to analyze the GSE110993 (miRNA) and GSE122709 (lncRNA and mRNA) data, resulting in 40 miRNAs, 1677 lncRNAs, and 5118 mRNAs. We also used the edgeR package to analyze the data, resulting in 62 miRNAs, 1313 lncRNAs, and 3748 mRNAs. The intersection function in R was used to identify the common differentially expressed lncRNAs between the results of DESeq2 and edgeR analyses. The common differentially expressed lncRNAs were considered to be differentially expressed lncRNAs (DELs) in ischemic stroke. Finally, we obtained 1275 DELs, 35 DEMis, and 3693 DEMs. The results are shown in Figure 1.

### 3.2. Construction of the lncRNA-microRNA-mRNA Network

**Figure 2:**
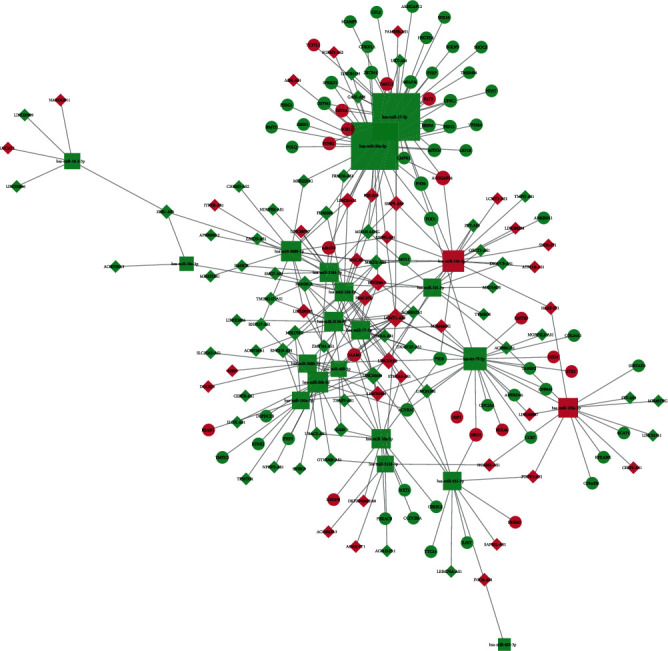
Visualization of the lncRNA-miRNA-mRNA network, which is composed of 91 lncRNA nodes, 70 mRNA nodes, 21 miRNA nodes, and 288 edges. The diamond nodes represent the lncRNAs, the circular nodes represent the mRNAs, and the square nodes represent the miRNAs. Red represents upregulated expression, whereas green represents downregulated expression. The size of the node represents the degree of the node.

We used Cytoscape to construct and visualize the lncRNA-miRNA-mRNA network for ischemic stroke, and the results have shown that the network consisted of 91 lncRNA nodes, 70 mRNA nodes, 21 miRNA nodes, and 288 edges. The result is shown in Figure 2.

### 3.3. GO Analysis and KEGG Pathway Analysis of lncRNAs

**Figure 3:**
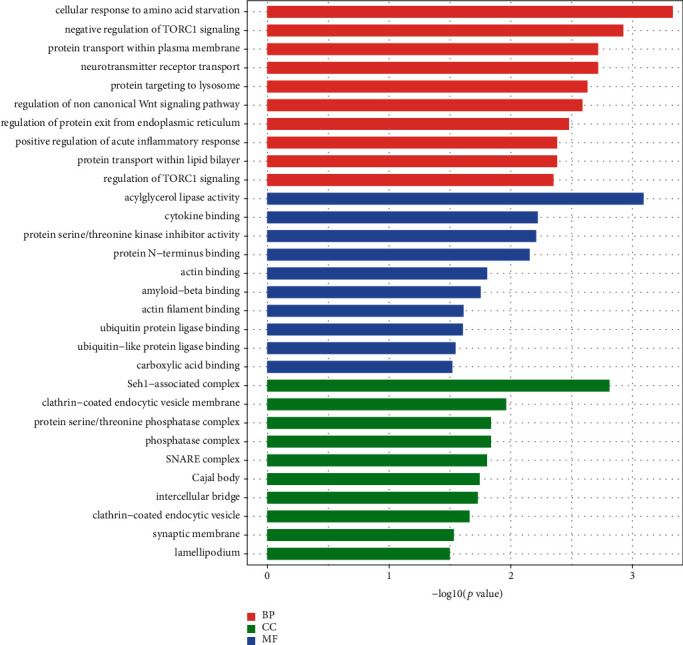
GO analysis of lncRNAs in ischemic stroke. Red represents the biological process, green represents the cell component, and blue represents the molecular function.

**Figure 4:**
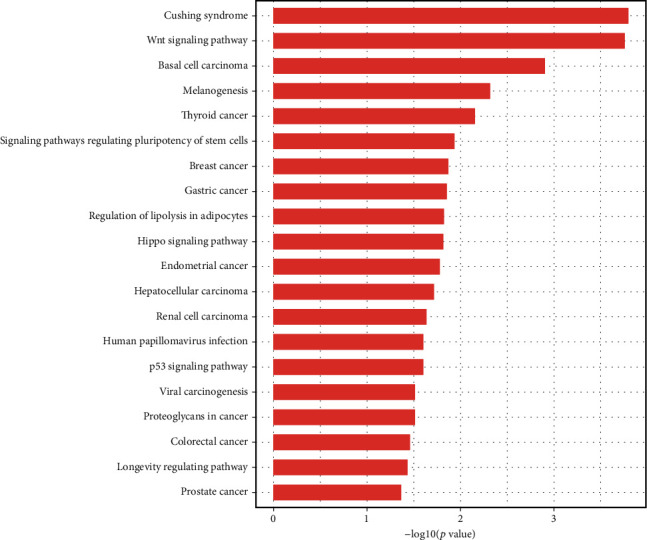
KEGG analysis of lncRNAs in ischemic stroke.

In the ceRNA network, the function of a lncRNA was predicted by exploring the function of the mRNA that it regulates. To understand the biological function of the lncRNAs in the ceRNA network of ischemic stroke, we performed GO analysis and KEGG pathway analysis of all DEMs in the network. GO analysis has shown that 191 terms were significantly enriched, and the main terms were as follows: (i) in “biological process,” whereby lncRNAs were mainly associated with the cellular response to amino acid starvation, negative regulation of TORC1 signaling, and neurotransmitter receptor transport; (ii) in “cell component,” whereby lncRNAs were mainly associated with the Seh1-associated complex, clathrin-coated endocytic vesicle membrane, and protein serine/threonine phosphatase complex; and (iii) in “molecular function,” whereby lncRNAs were mainly associated with the acylglycerol lipase activity, ubiquitin-like protein ligase binding, and cytokine binding. The GO analysis results are shown in Figure 3. KEGG pathway analysis has shown that 23 pathways were significantly enriched, and lncRNAs were mainly involved in the Wnt signaling pathway, human papillomavirus infection, p53 signaling pathway, and signaling pathways regulating pluripotency of stem cells. The results of GO analysis and KEGG pathway analysis are shown in Figures 3 and 4. For detailed information regarding the results of GO analysis and KEGG pathway analysis, please refer to Supplementary Material Tables S2 and S3.

### 3.4. Exploration of Key lncRNAs in Ischemic Stroke

**Figure 5:**
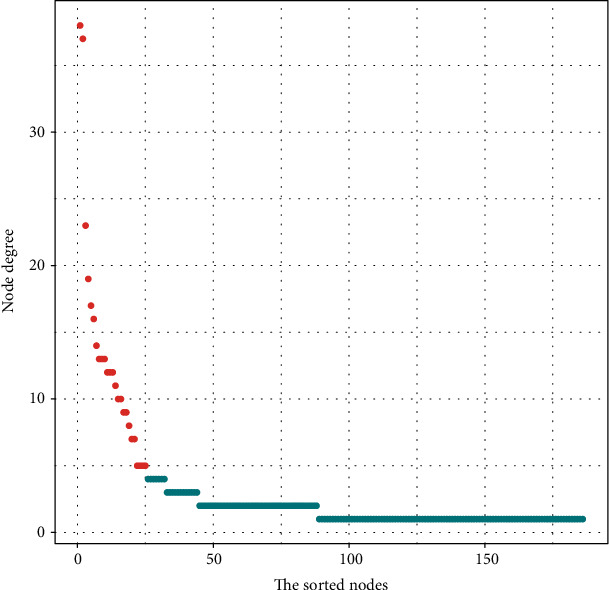
Node degree analysis of the nodes reveals specific properties of the lncRNA-miRNA-mRNA network. The abscissa axis represents the nodes sorted according to the node degree, the left side of the abscissa axis represents nodes with a high degree of nodes, and the right side of the abscissa axis represents nodes with a low degree of nodes. The ordinate axis represents the node degree of the node. The red dots represent the central node, and the blue dots represent the noncentral nodes.

Hub nodes form an important part of the ceRNA network, and node degree is considered to be a key element of a network [24]. We calculated the node degrees of all nodes in the lncRNA-miRNA-mRNA network. Finally, we identified 25 nodes as central nodes (node degree > 5), comprising 6 lncRNAs and 19 miRNAs.  Table 1 and Figure 5 show the 25 central nodes we found. In addition, we calculated the number of first relationship lncRNA-miRNA pairs and secondary relationship miRNA-mRNA pairs. Four lncRNAs had both higher node degrees and higher numbers of lncRNA-miRNA and miRNA-mRNA pairs (Table 2), indicating that these four lncRNAs are highly correlated with the occurrence of ischemic stroke. The details of the four lncRNAs are as follows: LCMT1-AS2 (LCMT1 antisense RNA 2), ERVH48-1 (endogenous retrovirus group 48 member 1), LINC01002 (long intergenic nonprotein coding RNA 1002), and LINC00638 (long intergenic nonprotein coding RNA 638).

### 3.5. Construction of lncRNA-miRNA-mRNA Subnetworks

We selected four key lncRNAs related to ischemic stroke, extracted the miRNAs and mRNAs related to them, and reconstructed the corresponding lncRNA-miRNA-mRNA subnetwork. The lncRNA ERVH48-1-miRNA-mRNA network was composed of 1 lncRNA node, 7 miRNA nodes, 41 mRNA nodes, and 48 edges. The lncRNA LCMT1-AS2-miRNA-mRNA subnetwork consisted of 1 lncRNA node, 12 miRNA nodes, 81 mRNAs, and 93 edges. The lncRNA LINC00638-miRNA-mRNA subnetwork consisted of 1 lncRNA node, 9 miRNA nodes, 17 mRNA nodes, and 26 edges. The lncRNA LINC01002-miRNA-mRNA subnetwork consisted of 1 lncRNA node, 7 miRNA nodes, 22 mRNA nodes, and 29 edges.

### 3.6. Verification of Key lncRNAs

**Figure 6:**
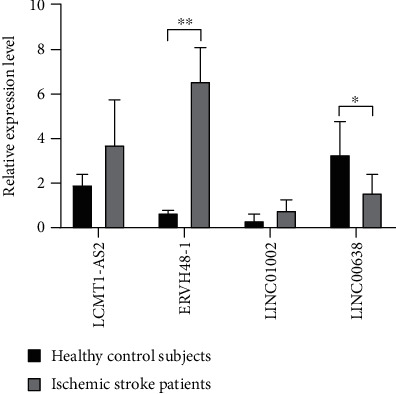
Comparison of the relative expression of lncRNAs between ischemic stroke patients and healthy control subjects. ^∗^*p* < 0.05 and ^∗∗^*p* < 0.01.

To verify the authenticity of the key lncRNAs we identified, we selected six blood samples from ischemic stroke patients and six blood samples from healthy control subjects for qRT-PCR molecular validation. The results have shown significant differences (*p* < 0.05) in the relative expression of two key lncRNAs (ERVH48-1 and LINC00638) between ischemic stroke patients and healthy control subjects, as shown in Figure 6.

## 4. Discussion

Stroke is a neurological disease with high rates of mortality and disability. The pathogenesis of stroke is acute focal injury of the central nervous system, leading to impaired neural function. Approximately 780,000 people worldwide experience stroke every year, with high rates of mortality and disability. Ischemic stroke accounts for the majority of cases. In the past decade, many studies have investigated the molecular mechanisms of ischemic stroke, focusing on mRNAs and miRNAs [25–28]. Recent studies have shown that lncRNAs have an important regulatory role in biological processes such as the regulation of transcription and the processing and splicing of mRNA. Abnormal expression of lncRNAs is associated with a variety of diseases, including neurological conditions. Although there have been many studies related to lncRNAs, there are still few studies on the key lncRNAs associated with ischemic stroke.

Salmena et al. proposed the ceRNA hypothesis, in which there are many types of endogenous ceRNAs, such as mRNAs, pseudogene transcripts, and lncRNAs [9]. There may be multiple MREs in a single ceRNA chain that can bind to different miRNAs, and each miRNA can silence multiple transcripts, resulting in a large, complex posttranscriptional regulatory network. LncRNAs can act as ceRNAs, binding to miRNAs through MREs to affect the protein expression of coding genes. Therefore, it is very important to study the function and regulatory mechanisms of lncRNAs as ceRNAs in ischemic stroke and to explore their potential significance in diagnosis. In this study, we downloaded gene expression data from GEO and used bioinformatics methods to analyze lncRNA, miRNA, and mRNA data in ischemic stroke. Based on the ceRNA theory, we constructed a lncRNA-miRNA-mRNA network for ischemic stroke, identified and validated key lncRNAs, and explored the molecular mechanisms of their regulation and development in ischemic stroke, providing a reference for molecular diagnosis and targeted therapy in the future.

To determine the function of lncRNAs in the ceRNA network of ischemic stroke, GO analysis and KEGG pathway analysis were performed on the mRNAs regulated by the lncRNAs. GO analysis has shown that 191 terms were significantly enriched, and the main terms were as follows: (i) in “biological process,” whereby lncRNAs were mainly associated with the cellular response to amino acid starvation, negative regulation of TORC1 signaling, and neurotransmitter receptor transport; (ii) in “cell component,” whereby lncRNAs were mainly associated with the Seh1-associated complex, clathrin-coated endocytic vesicle membrane, and protein serine/threonine phosphatase complex; and (iii) in “molecular function,” whereby lncRNAs were mainly associated with the acylglycerol lipase activity, ubiquitin-like protein ligase binding, and cytokine binding. “Cellular response to amino acid starvation” is a self-protective response of cells in the absence of amino acids. Amino acids can prevent excessive muscle catabolism in patients after stroke by inhibiting myofibrillar protein and skeletal muscle degradation. Stroke patients who lack amino acids have slower body function recovery and poorer prognosis [29]. In the cerebral ischemic environment, the PI3K/Akt/mTORC1 pathway is inhibited, protein synthesis is reduced, and autophagy is enhanced, leading to apoptosis [30]. In the acute inflammatory phase of ischemic stroke, neutrophil invasion is associated with the destruction of the local blood-brain barrier and the formation of infarction. The greater the number of neutrophils infiltrating ischemic hemispheres, the larger the infarct size [31]. In addition, Wang et al. found that the expression and activity of brain lipoprotein lipase increased after acute cerebral ischemia and reperfusion in rats [32]. The KEGG pathway analysis has shown that 23 pathways were significantly enriched, including the Wnt signaling pathway, human papilloma virus (HPV) infection, p53 signaling pathway, and signaling pathways regulating pluripotency of stem cells. Interestingly, it has been reported in the literature that these pathways play an important role in ischemic stroke. Liebner et al. found that the Wnt signaling pathway can maintain and protect the blood-brain barrier as well as damaged endothelial cells, thereby promoting recovery in ischemic stroke [33, 34]. In addition, Wu et al. proved that the HPV early cancer proteins E6 and E7 are specific tumor suppressor proteins that can prevent serine phosphorylation of p53 protein and protect the brain from apoptosis [35, 36]. Moreover, Jiang et al. found that when pluripotent stem cells were transplanted into the brain area damaged by stroke, they differentiated into neuron cells to promote the functional recovery of rats with stroke [37]. All of the above evidence shows that our results are very reliable and that these lncRNAs play an important role in ischemic stroke.

To identify key lncRNAs that could serve as targets for the diagnosis and treatment of ischemic stroke, the number of central node degrees and relationship pairs in the ceRNA network was analyzed. We identified four lncRNAs (LCMT1-AS2, ERVH48-1, LINC01002, and LINC00638) as key nodes, because their node degrees and numbers of lncRNA-miRNA and miRNA-mRNA relationship pairs were significantly higher than those of other lncRNAs. This suggests that these four lncRNAs have an important influence on the occurrence and development of ischemic stroke. Few studies have reported the function of these four key lncRNAs to date. We attempted to use the ceRNA theory to elucidate their molecular mechanisms in the development of ischemic stroke. We speculate that lncRNA S5645.1 may affect the expression of ischemic stroke target genes by competitively binding to miRNAs (such as those of the miR-17-5p and miR-20a-5p families). In support of our prediction, recent studies have shown that miR-17-5p and miR-20a-5p have important roles in the development of embolic stroke. In addition, miR-17-5p is an independent predictor of stroke, and its expression in the serum of patients with ischemic stroke is significantly higher than the average [38, 39]. LINC01002 and ERVH48-1, as ceRNAs, affect the expression of target genes of ischemic stroke by competitively binding to the hsa-let-7f-5p family. Regarding the lncRNA LINC00638-miRNA-mRNA network, studies have shown that miR-143-3p is a potential biomarker for acute ischemic stroke; it binds to LINC00638, has significantly higher expression levels in extracellular vesicles in patients with acute ischemic stroke, and is more specific than CT [15]. Finally, we used qRT-PCR to perform molecular verification of the four key lncRNAs. The expression levels of two key lncRNAs (ERVH48-1 and LINC00638) were significantly different between ischemic stroke patients and healthy control subjects. In future studies, we plan to collect a greater number of clinical samples to study the pathogenesis of ischemic stroke.

Compared with previous studies on ischemic stroke, this research has the following innovations: (1) use ceRNA theory to mine key lncRNA and (2) collect clinical samples to verify the authenticity of key lncRNA. Of course, our research also has some limitations. In this research, we only collected six clinical samples, which limited the verification of lncRNA. In future research, we plan to collect more clinical samples to study the pathogenesis of ischemic stroke.

## 5. Conclusions

In this study, we explored the key lncRNAs that influence the development of ischemic stroke from the perspective of epigenetics using bioinformatics methods. We found that four key lncRNAs (LCMT1-AS2, ERVH48-1, LINC01002, and LINC00638) may be highly associated with the pathogenesis of ischemic stroke. Then, we used qRT-PCR to perform molecular verification of the four key lncRNAs. The expression levels of two key lncRNAs (ERVH48-1 and LINC00638) were significantly different between patients with ischemic stroke and healthy control subjects. Finally, we think that these four lncRNAs are key lncRNAs of ischemic stroke and play important roles in the development of ischemic stroke.

Collectively, these findings will contribute to improve our understanding of the pathogenesis of ischemic stroke and the molecular mechanisms involved. We think that our work will contribute to the development of novel molecular targets, thus enabling early diagnosis, and targeted treatment of ischemic stroke patients.

## Figures and Tables

**Table 1:** List of differentially expressed miRNAs, lncRNAs, mRNAs.

Number	Gene name	Node degree	Gene type
1	hsa-miR-20a-5p	38	miRNA
2	hsa-miR-17-5p	37	miRNA
3	hsa-let-7f-5p	23	miRNA
4	hsa-miR-143-3p	19	miRNA
5	hsa-miR-26b-5p	17	miRNA
6	hsa-miR-3688-3p	16	miRNA
7	hsa-miR-125a-5p	14	miRNA
8	hsa-miR-18a-5p	13	miRNA
9	hsa-miR-3158-5p	13	miRNA
10	hsa-miR-3184-5p	13	miRNA
11	LCMT1-AS2	12	lncRNA
12	hsa-miR-126-5p	12	miRNA
13	hsa-miR-17-3p	12	miRNA
14	hsa-miR-532-5p	11	miRNA
15	hsa-miR-101-3p	10	miRNA
16	hsa-miR-3688-5p	10	miRNA
17	LINC00638	9	lncRNA
18	hsa-miR-193a-5p	9	miRNA
19	ERVH48-1	8	lncRNA
21	hsa-miR-3158-3p	7	miRNA
22	FAM157C	5	lncRNA
23	FAM201A	5	lncRNA
24	hsa-miR-16-2-3p	5	miRNA
25	hsa-miR-660-5p	5	miRNA

Note: mRNA: messenger RNA; miRNA: microRNA; lncRNA: long noncoding RNA.

**Table 2:** Number of lncRNA-miRNA and miRNA-mRNA pairs.

Number	Gene name	lncRNA-miRNA pairs	miRNA-mRNA pairs	Total number
1	LCMT1-AS2	12	81	93
2	ERVH48-1	8	41	49
3	LINC01002	7	22	29
4	LINC00638	9	17	26
5	FAM157C	5	9	14
6	FAM201A	5	0	5

Note: mRNA: messenger RNA; miRNA: microRNA; lncRNA: long noncoding RNA.

## Data Availability

GSE110993 and GSE122709 can be downloaded from the GEO database. URL of the GEO database is as follows: https://www.ncbi.nlm.nih.gov/geo/.

## Supplementary Materials

Supplementary 1 Table S1: detailed information regarding qRT-PCR primer of 4 key lncRNAs.

Supplementary 2 Table S2: detailed information regarding the results of GO analysis.

Supplementary 3 Table S3: detailed information regarding the results of KEGG pathway analysis.
